# Experimental investigation of earth-air heat exchanger using porous clay vessels for eco-friendly buildings

**DOI:** 10.1038/s41598-024-67212-5

**Published:** 2024-07-30

**Authors:** Emad M. S. El-Said, Gamal B. Abdelaziz, Mohamed I. M. Abdelhady, Nadia shokry, Sherif Mohamed, Mohamed A. Dahab

**Affiliations:** 1 Mechanical Engineering Department, Faculty of Engineering, Damietta University, Damietta, Egypt; 2 Mechanical Department, Faculty of Technology and Education, Suez University, P.O. Box: 43221, Suez, Egypt; 3 Department of Civil and Architectural Constructions, Faculty of Technology and Education, Suez University, P.O. Box: 43221, Suez, Egypt; 4 Ismailia Architectural School, Ismailia, Egypt

**Keywords:** Clay vessels, Ground heat exchanger, Evaporative cooler, Eco-friendly building, Geothermal energy, Environmental sciences

## Abstract

This study introduces an experimental investigation of a novel direct trend evaporative cooler based on a ground-air heat exchanger (GAHE) using porous clay vessels as an evaporation media under a variety of operational conditions, including air flow rate, inlet air temperature, temperature of inlet water, and in air humidity. The evaluation of the GAHE performance was based on the air-cooling effect, wet-bulb and dew-point efficiencies, energy efficiency ratio, water evaporation rate, specific water evaporation, specific cooling capacity, specific total cost, and CO_2_ emission rate. The influences of dry-bulb temperature, the incoming air's relative humidity (RH), and six air flow rates ranging from 11 to 25 L/s on the performance are investigated and discussed. Results indicated that increasing the air flow rate leads to an increase in the cooling capacity. Energy efficiency ratio (EER) reaches the highest value of about 25.5 recorded at 3:00 PM with air flow rate = 11 L/s. The lowest EER value is approximately 7.2 when the measured inlet and outlet temperatures are the closest at 7:00 PM, with a flow rate of 25 L/s. Increasing the air flow rate from 11 to 17 L/s increased the wet bulb efficiency, and the airflow rate was inversely proportional to wet-bulb efficiency. The maximum and minimum average dew-point efficiencies are 64% and 58% at 17 L/s and 22 L/s respectively. The water evaporation rate increases by 182.1%, increasing the air flow rate from 11 to 25 L/s.

## Introduction

Building energy requirements have significantly increased in recent years due to an expanding population and improved living conditions. About 33% of global energy consumption is used for space cooling and heating^1,2^. Due to the relatively high indoor air temperatures in the summer, air conditioning is mainly used for cooling, which results in considerable energy consumption in residential buildings. It is essential to look at alternative passive heating and cooling methods. A passive technology for cooling and heating is the GAHE. However, it provides advantages for the environment and the economy. It makes use of the thermal potential of the subterranean earth. A dependable soil depth has a steady temperature that serves as a winter and summer energy source and sink^3^. To optimize the design of GAHE, thermal performance testing is accurate and crucial.

The procedure considers the pipe's length, material, heat exchanger type, buried depth, and air flow rate through the pipe. Porous Clay Vessels (PCVs) for GAHE systems come in various designs and configurations, each tailored to suit specific applications and performance requirements. He et al.^4^ investigated the cooling effects of a porous, water-absorbent ceramic material for passive evaporative cooling. The passive cooling approach is intended to reduce summertime surface temperatures and establish cooler urban environments. The majority of research on clay as a membrane material focused on pillared clays^5–7^. Just recently, research on membranes made wholly from clay began^8,9^. The modification of membrane material and membrane surface has a significant effect on separation properties. The surface of the membrane material was modified to enhance its hydrophilicity. Typically, ceramic membranes were made from metal oxides such as alumina, zirconia, and titania, as well as organic clay powder constituted primarily of metal oxides. These materials are initially permeable due to surface hydroxyl (–OH) groups, which can readily link water molecules^10,11^. Extensive research has been conducted on the mechanism of heat and mass transport inside the planar membrane humidifier. Wang et al.^12^ studied a porous plate's heat and water transmission process within a fuel cell's self-humidifying system. The results demonstrated that the transfer of heat and transmission of water was increased by using a countercurrent flow and raising the humidifying gas's inlet temperature and RH. It is possible to simultaneously improve the water recovery rate by utilizing a countercurrent flow, raising the humidifying gas's intake temperature, and lowering the RH. Increasing the degree of the medium porosity can also facilitate water transfer. A heat and mass transportation model of evaporative refrigerators made of clay pots for storing vegetables was evaluated by Rehman et al.^13^. Results indicated that despite the boost in convection heating, an increase in local wind speed has a net beneficial impact on refrigeration efficiency.

Using porous clay vessels for GAHE with a focus on evaporative cooling is an intriguing application that combines traditional materials with innovative cooling techniques. Evaporative cooling takes advantage of the natural water evaporation process to cool the incoming air, making it an energy-efficient way to provide comfort in hot and dry climates^14^. Dogramacı and Aydın^15^ presented an Experimental comparison of evaporative cooling applications in arid climates. The combination of evaporative cooling with contemporary architecture was evaluated by Ibrahim et al.^16^. The Investigation employed porous clay materials as wet media, integrated with heat pipe heat exchangers; the supply air and operating air flows were staged in separate channels and in opposite directions. Results indicated that supply air would be chilled below the wet-bulb temperature to achieve a significant cooling effect and efficiency.

Using evaporative cooling to achieve thermal comfort in buildings was reviewed by^17–22^. Bora et al.^23^ presented the performance enhancement techniques of evaporative air coolers. Results revealed that Evaporative air coolers have been discovered to be superior to air conditioners.

Geothermal energy (GE) is an extensive and prospective renewable energy source for building cooling, ventilation, and heating^24^. Soni et al.^25^ reviewed the GE application integrated with GAHE. Globally, the GAHE systems technology is widely recognized. Lekhal et al.^26^ investigated the performance of a residential house integrated with solar floor and GAHE. Results indicated that the combination of solar floor and GAHE saved power needs for heating and cooling by 70, and 66%, respectively. The annual cooling energy saving of 9.6% for Houston and 13.8% for Dallas was analyzed by Do et al.^27^. The performance evaluation of GAHE in Egyptian claymate was investigated by Serageldin et al.^3^. The optimal depth for submerging GAHE pipelines is 3 m when the ground temperature is 32 °C in the summer and 29 °C in the winter^28^. Mathur et al.^29^ performed a Comparative study of straight and spiral GAHE systems operated in cooling and heating modes. The cooling and heating COP were 5.94 and 6.24 in summer 1.92 and 2.11 in winter, respectively. Soni et al.^30^ used GE to save the consumed energy for a 1.5 TR window air-conditioner. The evaluation of using GAHE under the climate condition of India was studied by Tiwari et al.^31^. Karabacak et al.^32^ investigated the cooling performance of the ground source heat pump system under the climates of Denizli, Turkey. Results revealed that the COP ranged between 3.1 and 4.8.

As a result of global warming, the electrical power consumption of cooling appliances is on the rise, increasing carbon emissions^33^. Earth contact cooling technologies are one of the potential solutions to this issue. Several investigations have been conducted on this topic. Ozgener and Ozgener^34^ conducted an exergoeconomic assessment of the Earth-Air Heat Exchanger (EAHE) system employed for greenhouse cooling in one of the research investigations on cooling. The investigation of a novel passive air conditioning system was performed by Li et al.^35^. The integration of EAHEs with solar collector-enhanced solar chimneys was their primary focus, to enhance the cooling system's overall efficiency. The system's ability to maintain indoor thermal comfort within a favorable range was demonstrated by the results, which demonstrated that the simultaneous augmentation of geothermal and solar energy can result in significant energy savings in the construction industry and reduce peak electricity demand during the summer. By incorporating an air supply static pressure chamber, Yang et al.^36^ mitigated temperature variations and enhanced cooling capacity. Their results indicated that the suggested technique, which employs EAHE, can effectively mitigate the temperature and variations in the outlet air, thereby offering a low-emission solution to enhance the thermal environment of structures. Li et al.^37^ demonstrated that implementing a sprinkler system in an EAHE increases COP and cooling capacity. They also developed a novel fresh air supply system that offers independent and dependable refrigeration with EAHE in their studies.

According to a review of all the cooling technique literature and an analysis of the current cooling techniques, the following findings were concluded:

- **Cooling efficiency**: Most studies report significant reductions in indoor temperatures, ranging from 5 to 18 °C, depending on system design and local climate conditions.
- **Energy savings**: Energy savings in HVAC applications are consistently reported, with reductions ranging from 25 to 30%.
- **Key Findings**: The consistent theme across studies is that GAHE systems, when optimized and integrated properly, significantly enhance energy efficiency and provide effective climate control in eco-friendly buildings.

In addition to that, an air conditioner's installation and operating costs are too high, and it also releases environmentally harmful greenhouse gases. On the other hand, the classic evaporative air cooler is less expensive than an air conditioner but has some cooling limits. Hence, these findings underscore the potential of GAHE systems with porous clay vessels in achieving sustainable and energy-efficient building designs. The current study aims to introduce a novel eco-friendly direct evaporative cooler based on GAHE using porous clay vessels as an evaporation media. The suggested system is examined to determine the influence of operating conditions on its performance, such as flow rate, temperature of inlet air, water temperature, and humidity of inlet air. As evaluation criteria, the metrics of air-cooling effect, wet-bulb and dew-point efficiencies, energy efficiency ratio, water evaporation rate, specific water evaporation, specific cooling capacity, specific total cost, and CO_2_ emission rate.

## Experimental work and measurement devices

### Experimental setup

**Figure 1:**
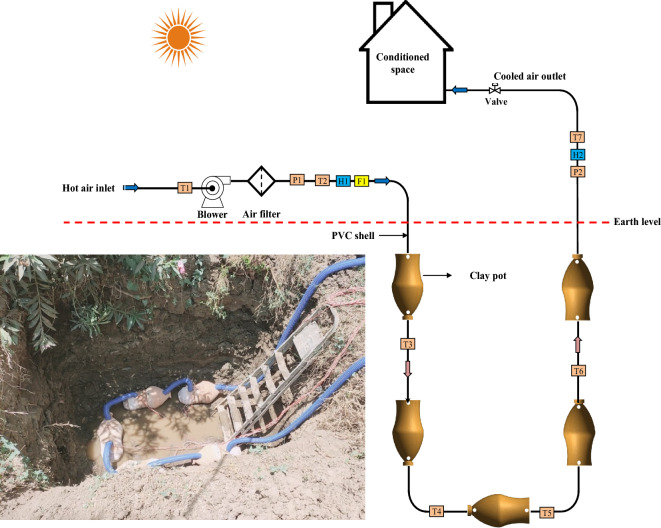
Schematic drawing and photographs of the suggested test rig.

**Figure 2:**
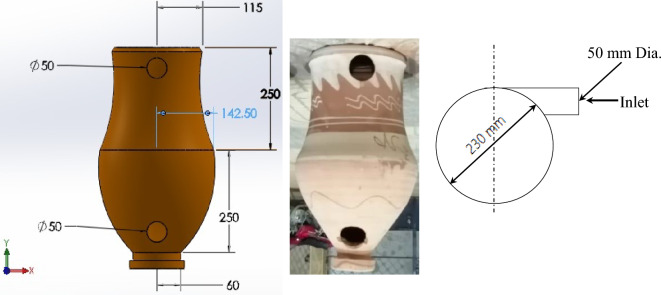
PCV and feeding method.

Figure 1 shows the schematic drawing and photographs of the suggested test rig utilized to evaluate the performance of porous clay vessels (PCV), while Fig. 2 shows a photograph of the PCV with dimensions. The ground heat exchanger was established and tested under the climates of Birkat Elsab City, Monufia, Egypt (Latitude of 30° 38′ 19.28'' N, Longitude of 31° 4′ 52'' E), and the system operates through 11 h, starting at 08:00 AM from 9 Sep. to 13 Oct. 2022. As shown in Fig. 1, the GAHE system consists of the ground and air supply circuits. The heat exchanger is composed of three burial parts with a 9 m total length of PVC pipe with five PCVs; 2 m vertical (inlet pipe) plus 5 m horizontal plus 2 m vertical (outlet pipe)) and inner diameter 0.0508 m (2 in.) (Table 1). The PCV geometry, which was used in all experiments and was built of very porous red clay with an 8 mm thickness, is shown in Fig. 2a in both a photograph and a schematic diagram. The upper cylindrical section and bottom frustum formed the PCV, which possessed cylindrical symmetry. Its total surface area, including the top and base, is 0.9 m^2^. Table 2 contains the properties of PCV. The PVC pipes were fixed with the clay vessel pipes with the help of circular holes at specific locations in U arrangement as presented in Fig. 1. The appropriate distance equal to 45 cm was maintained between the clay vessels for proper circulation of air inside the GAHE as shown in Fig. 1. Four bends connect the buried PVC pipes. Due to the vessel's porosity, a thin layer of water generated on the interior surface of the clay jar is used in the current experiment setup to interchange sensible and latent heat with the moving air. Therefore, a water-circulating pump, a vital element of any traditional evaporative air cooler, is unnecessary. As shown in Fig. 2b, A swirl tube uses a tube tangential entrance with fins to considerably improve the wall heat transfer by increasing the turbulent mixing close to the wall. Strong swirling flow is produced circumferentially by the swirl tube with fins.

**Table 1:** The main results summary of earth-air heat exchanger.

Authors	Depth of pipes (m)	Air flow rate (m3/h)	Specific energy gain (kWh/year)
Pfafferott^38^	2–4	12,000	13.5
Pfafferott^38^	2.3	1100	12.1
Pfafferott^38^	2	9000	23.8
Wagner^39^	1.5	3100	1.5
Burton^40^	2 m	3600–7200	–

**Table 2:** Properties of the PCV^41^.

Property	Units	Quantity
Density	kg/m3	2250
Specific heat	J/kg. °C	900
Thermal conductivity	W/m. °C	1.3
Porosity	%	0.775

The inlet end of the GAHE pipe connected to an air blower has an axial fan with a rated power 710 W, a maximum flow rate of 4 m^3^/min, and a top speed of 14,000 RPM, which is adjusted manually to manipulate air velocity. The airflow rate in these experiments varies from 11 to 25 l/s. The outlet end of the GAHE pipe is connected to a test room (conditioned space) with dimensions of 3.5 m × 3.5 m × 3 m (L × W × H), its roof and walls are thermally insulated and fully exposed to the open environment. A trough with dimensions of 1.5 × 2 × 2 m^3^ is dug. The GAHE was buried at a depth of 2 m, as shown in Fig. 1. To ensure better and uniform performance and avoid pores clogging of the porous media material by impurities in the water, after installing the pipes, the removed soil will be replaced with Loamy sand as an isotropic soil with homogeneous thermal conductivity.

### Measurement devices

In Fig. 1, F, T, H, and P demonstrate the locations for measuring flow rate, temperature, RH, and pressure. Airflow velocity is measured with a flow meter. At the inlets, between the PCVs, and at the outlets, sensors were installed to measure temperature and humidity to evaluate the heat and water transfer through the GAHE. In the experimental runs, the ambient air temperature and relative humidity (T1 and RH1) were recorded by instruments having 0.2% and 3% (full scale, FS) using a thermometer and a Hygrometer (HMT330), respectively. Sensor T1 simultaneously measures and logs the ambient temperature (*T*_*am*_). T2 and T7 thermocouples were placed at the inlet and outlet of GAHE. Four temperature sensors, T3 to T6, were mounted at a depth of 2 m from the ground surface and fixed with the assistance of PVC pipes to measure the time-varying air temperature, as depicted in Fig. 1. To measure the airflow rate (F1), a Rotameter was installed in series with the airflow line. The control of the discharge rates was accomplished by varying their speeds. Saturated, moist air is expelled from the test section. In addition to the ambient air temperature and RH, the efficacy of the characteristics of the test section at different operating conditions was also evaluated using these variables. Before determining the sensitivity of the probe, all sensors were calibrated. A graduation pressure gauge decided the pressure difference between the air inlet and exhaust. Table 3 presents the comprehensive technical details of the sensors and probes.

**Table 3:** Sensor and probe technical details.

Sensor-Type	Ref	Detail	Accuracy
Thermocouple K-type	Zhejiang	Temperature	± 1%
HTC-2 (− 50– + 70 °C) and (10%-99% RH)	Selectech	Temperature and Humidity	± 1%
DS 120	MGE	Solar radiation	± 1%
LZs-125 (15–150 m^3^/h)	SENTEC	Rotameter	± 4%
UT-212	Telenin	Pressure gauge	± 1.8%

The following steps are the experimental testing apparatus operating: (i) run the air blower at a pace that discharges the air flow rate required; (ii) measure the ambient and flowing air temperatures and RH reaching steady conditions; (iii) record the temperatures of flowing air and water and RH at steady state.

All case studies tested conditions are shown in Table 4.

**Table 4:** Test case conditions.

Date	Air flow rate (L/s)
13–10-2022	11
12–10-2022	13
10–10-2022	17
08–10-2022	20
07–10-2022	22
09–09-2022	25

## Performance assessment

In the investigations, the following parameters were measured hourly (each 1 h).

- The ambient temperature
- The temperature of the inlet and exit flowing air.
- The relative humidity of air at the inlet and outlet flow rate: RH1 and RH2.
- Ground temperatures: T3 to T6.
- Solar radiation.
- Pressure loss in GAHE.

When considering the performance of a direct evaporative cooler, the temperature of the air output, the pressure drop, and the cooling efficiency (*η*_*co*_) are the primary factors considered. The energy efficiency ratio (EER) is dependent on the pressure decrease through PCV, which is proportional to the consumed power required to operate the fan.

### Thermal analysis

Wet-bulb efficiency ($\eta_{wb}$) is the ratio of the temperature difference between the inlet and outlet of cooling air to the temperature difference between the inlet and moist bulb of cooling air^42^. The data measured, such as the inlet and exit air temperatures, determines the heat transfer efficiency in the absence of condensation in the primary air stream. The wet bulb efficiency $\eta_{wb}$ is defined by Eq. (1):

1 $$\eta_{wb} = \frac{{T_{d,in} - T_{d,out} }}{{T_{d,in} - T_{wb,in} }}$$

where *T*_*d,in*_ and *T*_*d,out*_ are the temperatures of dry channel inlet and outlet air, and *T*_*d,wb*_ is the temperature of wet-bulb air entering the dry channel.

On the other hand, the dew point effectiveness is the ratio of the difference between ambient air temperature and its dew point temperature. Mathematically, the expression for dew-point efficiency is as^42^:

2 $$\eta_{db} = \frac{{T_{d,in} - T_{d,out} }}{{T_{d,in} - T_{dp} }}$$

*Q*_c_, the cooling capacity.

Equation (3) determines the sensible cooling of GAHE^43^:

3 $$Q_{c} = C_{p,a} \dot{m}_{a} \Delta T$$

where *C*_*p,a*_ is the specific heat of air and $\dot{m}_{a}$ is the flow rate of air in kg/s.

The air temperature drop ($\Delta T$) or cooling effect was determined by considering the inlet and exit temperatures of the flowing air, as shown in Eq. (4)^8^:

4 $$\Delta T = \left( {T_{d,in} - T_{d,out} } \right)$$

The air blower power consumption *W*_*blower*_ is the only power supplied to a GAHE. The total power (*W*_*T*_) is determined by Eq. (5)^44^:

5 $$W_{T} = W_{blower} = \frac{{\dot{m}_{a} }}{{\rho_{a} \eta_{blower} }}\Delta p$$

where *Δp* is the pressure loss across the GAHE and *η*_*blower*_ is the fan motor efficiency which is assumed as 0.9^45^.

The industry devised the energy efficiency ratio (EER) to evaluate the energy consumption rate of air conditioning units. EER is determined by dividing the input electrical power (in watts) by the quantity of cooling produced (in British Thermal Units or BTU) under a single set of conditions^46^:

6 $$EER = \frac{{3.412 \times Q_{c} }}{{W_{T} }}$$

As shown in Eq. (7), the water evaporation rate (WER) in the GAHE system is calculated by multiplying the mass flow rate and absolute humidity difference of air (Δ*ω*).

7 $$WER = \dot{m}_{w} = \dot{m}_{a} \left( {\omega_{a,out} - \omega_{a,in} } \right)$$

Cooling capacity (*Q*_c_), specific cooling capacity (*SCC*), and specific water evaporation (*SWE*) can be calculated from^47^:

8 $$SCC = \frac{{Q_{c} }}{{\dot{m}_{w} }}$$

9 $$SWE = \frac{{\dot{m}_{w} }}{{\Delta T_{lm} A_{s} }}$$

where $\Delta T_{lm}$ is logarithmic mean temperature difference.

10 $$\Delta T_{lm} = \frac{{\left( {T_{d,in} - T_{wb,in} } \right) - \left( {T_{d,out} - T_{wb,out} } \right)}}{{ln\left[ {\frac{{\left( {T_{d,in} - T_{wb,in} } \right)}}{{\left( {T_{d,out} - T_{wb,out} } \right)}}} \right]}}$$

Energy's quantity is not as valuable as its quality, or the work that can be accomplished with a given level of energy, which is referred to as exergy (available work or availability). Exergy is the maximum quantity of work that a subsystem can perform as it reaches equilibrium with its surroundings in a reversible process, defined from a thermodynamic perspective^48^. Based on the second law of thermodynamics, the exergy analysis encompasses the entire exergy inflow, exergy outflow, and exergy destructed from the system. The general exergy balance of a system can be expressed as^49^:

11 $$\dot{E}x_{i} - \dot{E}x_{o} = \dot{E}x_{d,GAHE}$$

Considering the same control volume as for the energy Eq. (11) becomes^50^:

12 $$\dot{E}x_{{\text{heat }}} + \dot{E}x_{{\text{work }}} + \dot{E}x_{mass,i} - \dot{E}x_{mass,o} = \dot{E}x_{d,EAHE}$$

Substituting each exergy term by its corresponding formula, (12) becomes^50^:

13 $$\left( {1 - \frac{{T_{0} }}{{T_{w} }}} \right)\dot{Q} + \dot{W}_{mec} + \dot{m}\psi_{i} - \dot{m}\psi_{o} = \dot{E}x_{d,EAHE}$$

*T*_o_ is the reference temperature, which is the mean temperature of the selected environment. Tw is the tube wall temperature, determined by the median of the three wall temperature readings obtained along the tube. Note that in other investigations, the wall temperature Tw was measured at a single point^51^ or determined by utilizing the constant temperature of undisturbed soil^52^.

The mechanical power delivered by the fan to the air is defined by.

14 $$\dot{W}_{{\text{mec }}} = \dot{W}_{{\text{electric }}} \cdot \eta_{fan}$$

*ψ*_*i*_ and *ψ*_*o*_ are the specific flow exergies, which are determined using the humid air flow exergy formulated by^53^:

15 $$\begin{array}{*{20}c} {\psi_{n} = \left( {C_{p,a} + \omega_{n} \cdot C_{p,v} } \right)\left( {T_{n} - T_{0} } \right) - T_{0} \cdot \left( {C_{p,a} + \omega_{n} \cdot C_{p,v} } \right) \cdot \ln \left( {\frac{{T_{n} }}{{T_{0} }}} \right)} \\ { + T_{0} \cdot \left( {R_{a} + \omega_{n} \cdot R_{v} } \right) \cdot \ln \left( {\frac{{P_{n} }}{{P_{0} }}} \right) + T_{0} \cdot \left( {R_{a} + \omega_{n} \cdot R_{v} } \right) \cdot \ln \left( {\frac{{1 + 1.6078\omega_{0} }}{{1 + 1.6078\omega_{n} }}} \right)} \\ { + T_{0} \cdot 1.6078 \cdot \omega_{n} \cdot R_{a} \cdot \ln \left( {\frac{{\omega_{n} }}{{\omega_{0} }}} \right)} \\ \end{array}$$

Such that *n* is any point along the flow and *T*_0_, *P*_0_, and *ω*_0_ are the reference values of the temperature, pressure, and humidity ratios, respectively.

Based on its capabilities, exergetic efficiency is employed to determine a more accurate evaluation of the system. : The exergetic efficacy of the entire EAHE system is determined by:

16 $$\eta_{ex} = \frac{{\dot{E}x_{o} }}{{\dot{E}x_{i} }} = 1 - \frac{{\dot{E}x_{d} }}{{\dot{E}x_{i} }}$$

The rapid depletion of resources is a consequence of the utilization of power and the advancement of technologies. The concept of sustainability guarantees the equitable utilization of resources without jeopardizing the future. A performance measure and comparison tool is the sustainability index (SI)^54^. The following is the method used to calculate the index:

17 $$SI = \frac{1}{{1 - \eta_{ex} }}$$

### Economic analysis

For economic analysis, the initial and operating costs of the cooler are estimated for GAHE cooler. GAHE's primary components are a heat exchanger and an airflow fan. The entire cost of the GAHE is the sum of its operating and capital costs (initial costs). The total cost is computed as follows:

18 $$C_{T} = C_{op} + C_{main} + C_{c}$$

where *C*_*op*_ is the operating cost; it includes energy, operating personnel, and raw materials management. *C*_*main*_ is the maintenance cost and consists of technicians, maintenance facility costs, testing supplies, support for maintenance and processing costs, and maintenance spares and repair parts. *C*_*c*_ is the capital (initial) cost; the price of each component is shown in Table 4.

The costs are incurred in Egypt and converted to USDs using the current exchange rate. The USD is a universally recognized currency, which assists readers from all over the world in comprehending the economic analysis of GAHE. The cost of operation is determined by the amount of energy (electricity) required to operate the air turbine. The electricity consumption fee is estimated to be 0.039 USD/kWh globally. The operating cost of the system is computed based on 10 working hours daily for one year. The specific total cost (STC) is the total cost per unit cooling rate^55^ (Table 5).

19 $$STC = \frac{{C_{T} }}{{Q_{c} }}$$

**Table 5:** Cost of capital expenditures for GAHE, including installation expenses.

Item description	Unit	Unit price (USD)	Qty	Price (USD)
Pottery vessels	No	3	5	15
Air blower	No	20	1	20
Pipes, fittings	m	1	9	9
Excavation and filling costs	m3	5	6	30
Measuring and control devices	–	–	––	25
Installation cost	–	–	––	15
Accessories	–	–	–	6
Total cost	120	–	–	–

### Environmental analysis

Energy extraction from traditional sources has adverse environmental effects, including ozone depletion, acid rain, and carbon emissions. Electricity is utilized to power the fan and compressor. Coal is burned in electric substations to generate electricity. The carbon emission factor can be used to estimate the carbon emissions (CE) associated with electricity production. The CO_2_ emission is calculated by^56^,

20 $$CE = m_{{{\text{CO}}_{2} }} = \frac{{f_{{{\text{CO}}_{2} }} W_{{\text{blower }}} t_{{\text{Op }}} }}{{10^{6} }}$$

where $m_{{{\text{CO}}_{2} }}$ is the mass by kg of carbon and $f_{{{\text{CO}}_{2} }}$ is the emission factor for carbon. This factor varies from country to country based on the electricity generation methodologies. The carbon emission factor for Egypt is 0.5 kgCO_2_/kWh^57^. The *W*_*blower*_ is the power consumption throughout operating hours, measured in kilowatts, and *t*_*Op*_ is the device's operating hours, measured in yearly hours.

### Uncertainty analysis

For any measured or calculated parameter (*y*) as a function of independent variables $x_{1} ,x_{2} ,x_{3} , \ldots \ldots \ldots \ldots .,x_{n}$, $y = f\left( {x_{1} ,x_{2} ,x_{3} , \ldots \ldots \ldots \ldots .,x_{n} } \right),$ the uncertainty value (*u*) was calculated as follows^58^:

The uncertainty (*u*_*y*_) can be determined as follows:

21 $$u_{y} = \left[ {\left( {\frac{\partial y}{{\partial x_{1} }}u_{1} } \right)^{2} + \left( {\frac{\partial y}{{\partial x_{2} }}u_{2} } \right)^{2} + \left( {\frac{\partial y}{{\partial x_{3} }}u_{3} } \right)^{2} \ldots \ldots \ldots \ldots } \right]^{\frac{1}{2}}$$

where *u*_1_, *u*_2_, ….. are the uncertainties in $x_{1} ,x_{2} ,x_{3} , \ldots \ldots \ldots \ldots .,x_{n}$ As presented in Table 6.

**Table 6:** Uncertainty values.

Parameter	Value
Temperature	± 0.43 °C
Cooling effect	± 0.43 °C
EER	± 7. 6%
Wet-bulb efficiency	± 5.4%
Dew-point efficiency	± 0.83%
Specific cooling capacity	± 2.4%
Specific water evaporation	± 2.3%
Exergy efficiency	± 3.7%
Sustainability index	± 3.7%

## Results and discussions

**Figure 3:**
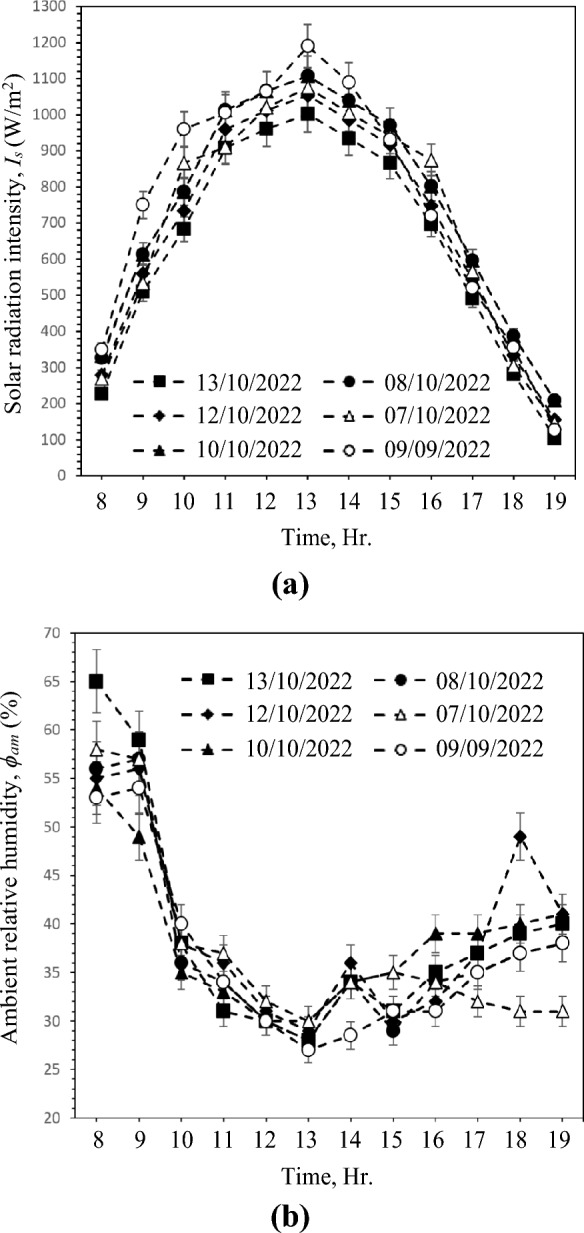
Hourly variation of (**a**) Solar radiation intensity and (**b**) Ambient RH.

There were six investigations conducted with airflow rates ranging from 11 to 25 L/s. The performance factors are evaluated using the values of air DBT at the outflow of the GAHE, outlet RH, and blower energy consumption. In addition, three major influences are investigated and discussed: dry-bulb temperature of the incoming air, RH of the incoming air, and airflow rate. Figure 3 presents hourly variation of solar radiation intensity and ambient RH. This Fig. shows that the values of the inlet air conditions depend on the solar radiation intensity. For all test cases, Fig. 3a shows that the solar radiation intensity reaches the maximum values at 13:00 PM by about 1190 W/m^2^ when the air flow rate is 25 L/s. Also, Fig. 3b illustrates that the maximum values of RH are in the morning at 08:00 AM by about 65% and minimum at 13:00 PM by 27%. These findings prove the dependency of humidity values and temperature on solar intensity.

### Cooling effect

**Figure 4:**
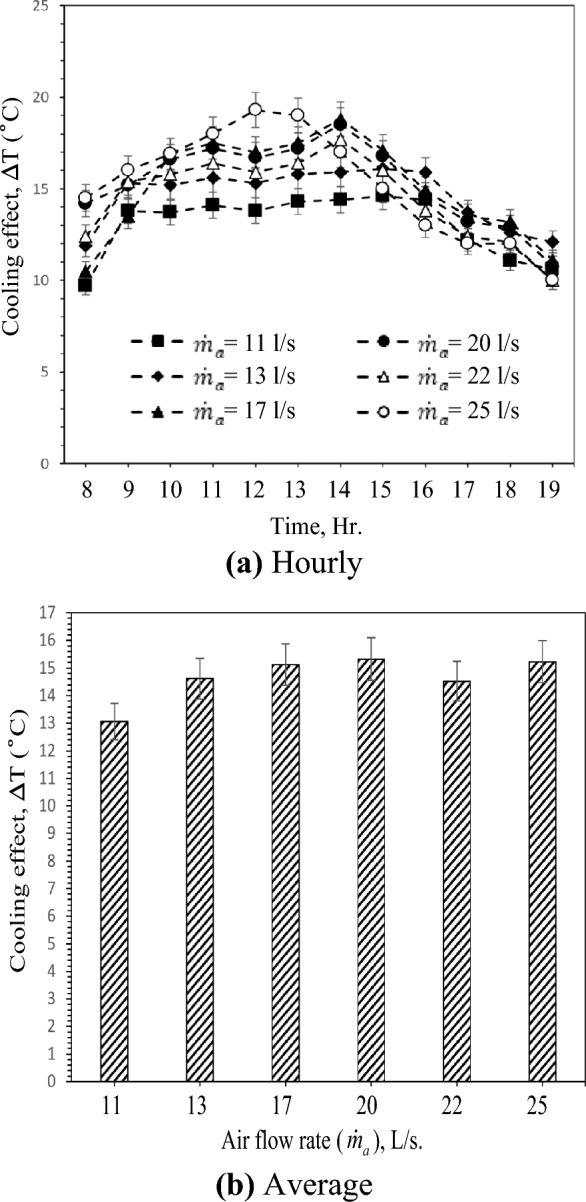
Hourly and average variation of cooling effect with different air flow rates.

The effect of porous clay vessels on the air-cooling impact was analyzed. Figure 4 depicts the average air temperature difference between the inlet and outlet of the test section of the porous clay vessels as a function of the air flow rate of 11L/s to 25L/s. Due to forced convective heat and mass transfer between clay vessels and moving air, the clay vessels' cooling effect is responsible for the temperature decrease across the test section. According to the proposed system design and structure and related heat and mass transfer processes, It is anticipated that as air mass flow rate increases, temperature decline will increase, most likely due to the increasing rate of water evaporation with the increase in airflow rate and the decreased retention time of air and clay vessel surface contact. However, with a high airflow rate, the clay vessels are predicted to get saturated in a shorter time; thus, the air temperature reduction could last shorter at high air flow rates. Also, a portion of air will pass by the clay containers without making contact, resulting in decreased water evaporation. On the other hand, the cooling impact of GAHE depends on the rate of evaporation and the internal conditions of the cooler. Temperature and humidity of incoming air have the greatest environmental impact on evaporation. Therefore, when the inlet temperature is high enough, significant evaporation can occur, and when the inlet RH is low enough, more water vapor can be absorbed by the air. According to Fig. 3, inlet temperature and air humidity vary depending on the solar radiation intensity. In Fig. 4a, the temperature difference between GAHE inlet and exit with a flow rate of 25 L/s shows the highest value of about 19.3 °C at solar radiation intensity = 1056 W/m^2^ and inlet RH = 30%, as shown in Fig. 3. Also, Fig. 4a demonstrates that the temperature gradient is high at a modest air flow rate of 11L/s and decreases as the airflow rate increases. The inlet air wet-bulb depression (WBD) is the difference between DBT and WBT at the inlet. It may significantly affect the exit air temperature. The cooling effect increases with the increase of inlet air temperature and vise versa.

In addition, as the temperature difference between the vessels' interior and exterior (soil) increased, the sensible heat exchange increases and the exit air temperature decreases. This result indicated that the system is ideally suited to warmer climates. The hourly cooling effect varied between 9.7 and 14.6 °C, 11.9 and 16.1 °C, 10.5 and 18.8 °C, 14.2 and 18.5 °C, 12.4 and 17.7 °C, and 14.5 and 19.3 °C, for the air flow rates of 11, 13, 17, 20, 22, and 25 L/s, respectively. The average cooling effect variation for different air flow rates is demonstrated in Fig. 4b. Cooling effect values (ΔT) 13.1 °C at 38.9% of inlet air average relative humidity, 14.6 °C at 39.2% of R.H., 15.1 °C at 38.3% of R.H., 15.3 °C at 37.3% of R.H., 14.5 °C at 37.4% of R.H., and 15.2 °C at 36.5% of R.H. for the air flow rates of 11, 13, 17, 20, 22, and 25 L/s, respectively. Furthermore, without the consideration of cooling capacity, decreasing the air flow rate reduced the exhaust air temperature, decreased the fan's energy consumption, and made the system more energy-efficient. Due to the reduced RH, the moisture on the wet surface was more likely to evaporate into the secondary air, resulting in a significant increase in latent heat transfer. Therefore, the proposed system has a more effective cooling effect in an environment with low RH. In other words, the cooling and dehumidification process via the suggested system can reduce the exit air temperature during hot and humid summer in hot arid climates.

### Energy efficiency ratio (EER)

**Figure 5:**
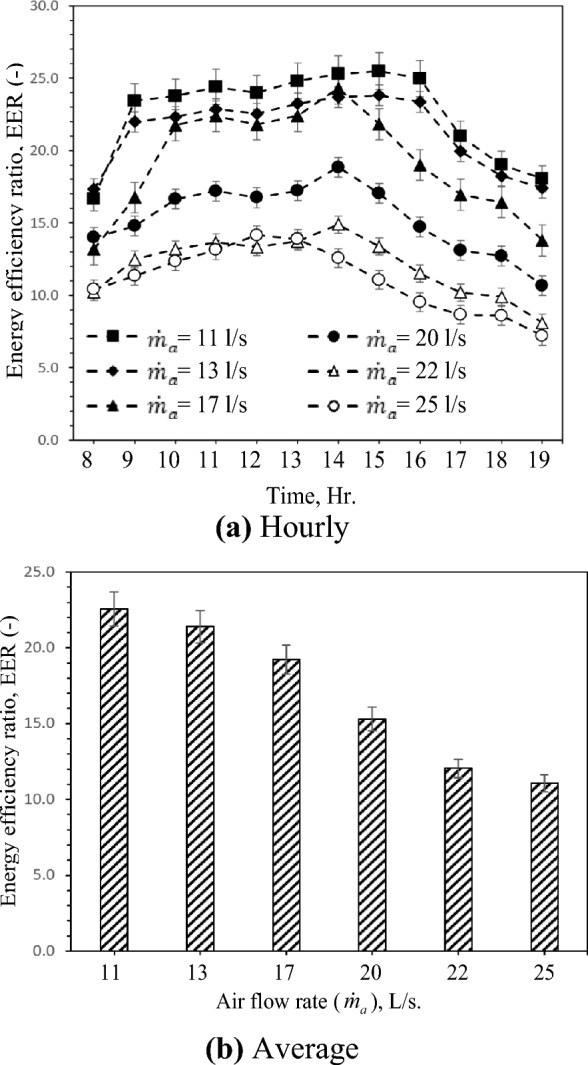
Hourly and average variation of EER with different air flow rates.

EER is the ratio between the cooling capacity and the fan power. It varies based on the heat transfer rate variation, which varies with ambient temperature. Figure 5 shows the hourly and average variation of EER with different air flow rates. EER is affected by operating parameters such as inlet air temperature and RH, solar radiation intensity, and ventilation rate. As stated in the preceding section, increasing the air flow rate increases cooling capacity. Increasing the air flow rate simultaneously increases the fan's output. Figure 5 a depicts the increase in EER with increasing solar radiation intensity and inlet air temperature, followed by a decrease in EER as solar radiation intensity increases towards the end of the day. This indicates that before 10:00 AM, the percent increase in cooling capacity with solar radiation intensity is more significant than the percent increase in fan power with solar radiation intensity and inlet air temperature due to the effect of heat added to the air viscosity and vice versa after 14:00 PM. Figure 5b demonstrates that the EER improves as the flow rate rises for all test cases. This can be ascribed to the increment of the cooling capacity with the increment of the airflow rates, which is a percent higher than the percent of the rise of the fan power. This variation is depicted in Fig. 5a, where EER reaches the highest value of approximately 25,5 at 15:00 PM with an airflow rate of 11 L/s. The lowest EER value is about 7.2 when the inlet and outlet temperatures are measured to be the closest at 19:00 PM with an airflow rate of 25 L/s. In general, *EER* increases between 09:00 AM and 16:00 PM and reaches its maximum at 14:00 PM as the ambient temperature is maximum related to high soil and water temperatures.

The inverse occurs during the remainder of the day when the EER reduces to its lowest point. When ambient temperature variation is considerable, EER variation is also high. As shown in Fig. 5b, the highest and lowest average EER values that can be attained at 11 L/s and 25 L/s air flow rate are 25.6 and 11.7, respectively, i.e., the EER increased by 103.8% when 14 L/s decreased the air flow rate. This is due to the increment and reduction in cooling capacity as the air and water inlet temperatures rise and reduce, respectively.

### Wet-bulb or cooling efficiency

**Figure 6:**
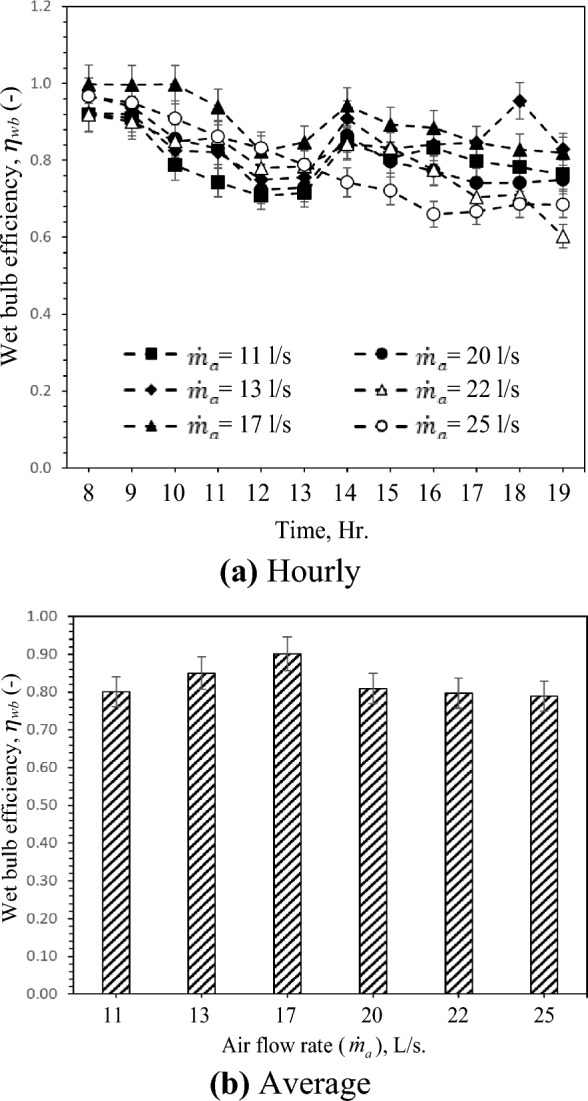
Hourly and average variation of wet-bulb efficiency with different air flow rate.

As presented in the above sections, The temperature of the air drops and the RH rises as it passes through the clay vessels. It is worth noting that the cooling effect with different air flow rates, as an essential and direct parameter to evaluate the evaporative coolers performance, is not enough to assess the suitability of the proposed system in different regions reasonably due to the different meteorological conditions of the test days. Thus, the regional adaptability of GAHE can be quantitatively analyzed by a performance parameter such as wet bulb efficiency, which is affected more by air humidity than other air parameters. Figure 6 shows the performance evaluation of the GAHE based on wet bulb or cooling efficiency. From Fig. 6a, the results indicate that GAHE has the highest wet-bulb efficiency ${\eta }_{wb}$ of 0.95 for 13 L/s air flow rate. Theoretically, if the process is considered adiabatic saturation and the test section is wholly insulated, this efficiency should be 100%. Figure 6 a demonstrates that the efficiency of a wet bulb will exceed 95%. This indicates that the actual process differs slightly from the adiabatic saturation process, particularly at higher inlet water temperature (from the heated soil at 18:00) and low airflow rate. With the increase of inlet air WBD, ${\eta }_{wb}$ Increases, as concluded in Fig. 6a,b indicate that with increasing the air flow rate from 11 to 17 L/s, wet bulb efficiency increased, and then the airflow rate was inversely proportional to wet-bulb efficiency. It is due to high heat and mass transfer between air to water and low-pressure drop until 17 L/s air flow rate with high retention time at lower air flow rate.

### Dew-point efficiency

**Figure 7:**
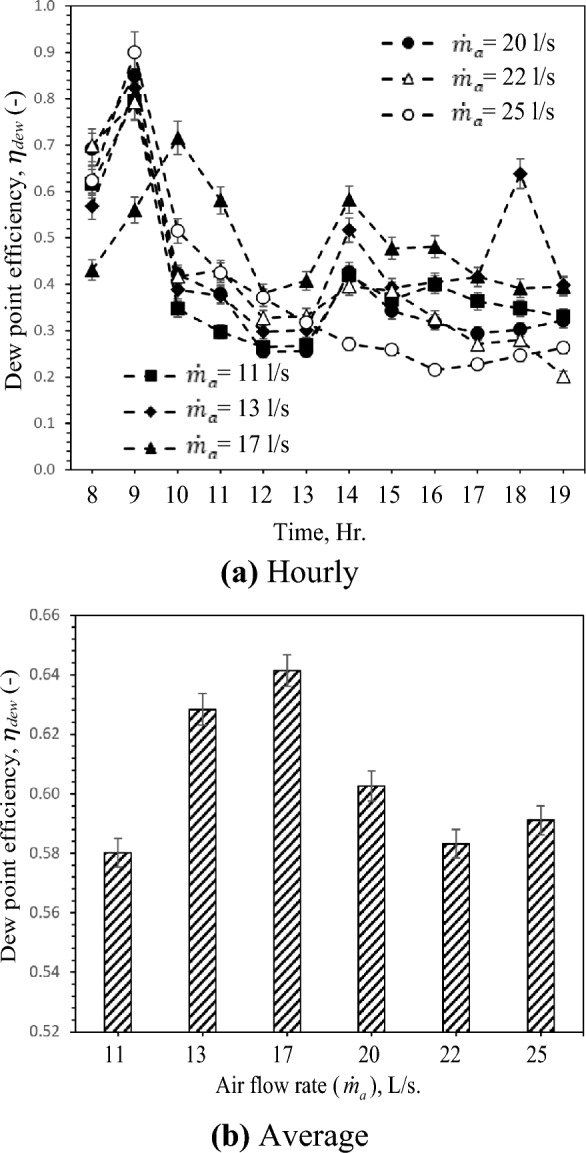
Hourly and average variation of dew-point with different air flow rates.

The dew point efficiency is determined using Eq. 2 based on air temperatures. From Fig. 7, it can be observed that the dew point efficiency varies qualitatively with the air temperatures approximately as wet bulb efficiency. Based on solar radiation intensity values and the varying inlet air temperatures in the experiment, the dew point efficiency ranges between 40 and 82% as shown in Fig. 7a. Depending on air humidity, the dew point efficiency varies with the increased inlet air temperature for all test cases. The evolution of the average dew point efficiency is shown in Fig. 7b as a function of the airflow rate. Inspection of this Fig shows that the dew point efficiency increases gradually with the increase of the airflow rate, and it is more pronounced at 17 L/s. In fact, the increment of the airflow rate, as discussed above, enhances evaporative cooling. After airflow reaches 17 L/s, this Fig shows the dew-point efficiency decreasing with the increase of the airflow rate in three rest flow rate values: 20 L/s, 22 L/s, and 25 L/s. This is because the inlet air parameters such as temperature and humidity and water temperature according to soil temperature play a critical role in temperature reduction affecting dew-point efficiency. The maximum and minimum average dew-point efficiency are 64% and 58% at 17 L/s and 22 L/s respectively.

### Water evaporation rate (WER)

**Figure 8:**
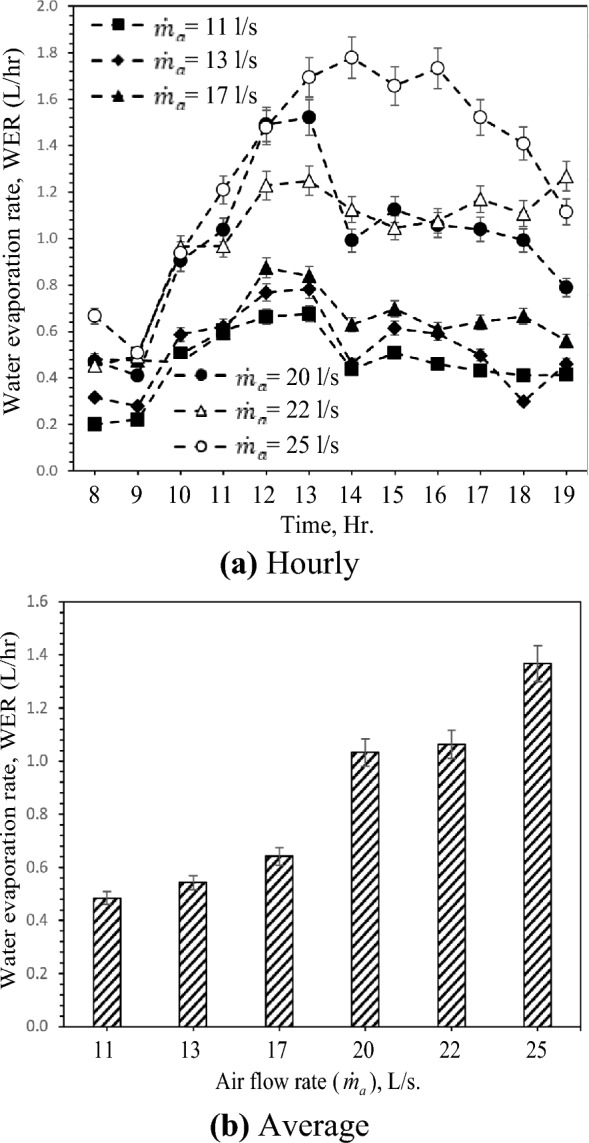
Hourly and average variation of water evaporation rate with different air flow rates.

As mentioned in previous sections, the evaporative cooling process involves mainly the cooling of air by means of rejecting the heat through the evaporation of water. The water evaporation rate (WER) increased significantly with increasing inlet air and water temperature across all experimental test cases. Still, this increase is faster between 09:00 AM and 14:00 PM, with the solar radiation intensity and soil temperature growth in this period. Then WER decreased gradually with decreasing inlet air temperature based on solar radiation intensity, as shown in Fig. 8a,b also indicates that the increase of the WER and the evaporation rate increased the airflow rate. These can be ascribed to the increment in air temperature drop and humidity increase. Also, the mass transfer coefficients are a function of air flow rate and air inlet temperature. Similarly, the evaporation rate increases by increasing the air flow rate and air inlet temperature. One can expect from Fig. 8b that the water evaporation rate rises by 182.1% by raising the air flow rate from 11 L/s to 25 L/s.

### Specific water evaporation (SWE)

**Figure 9:**
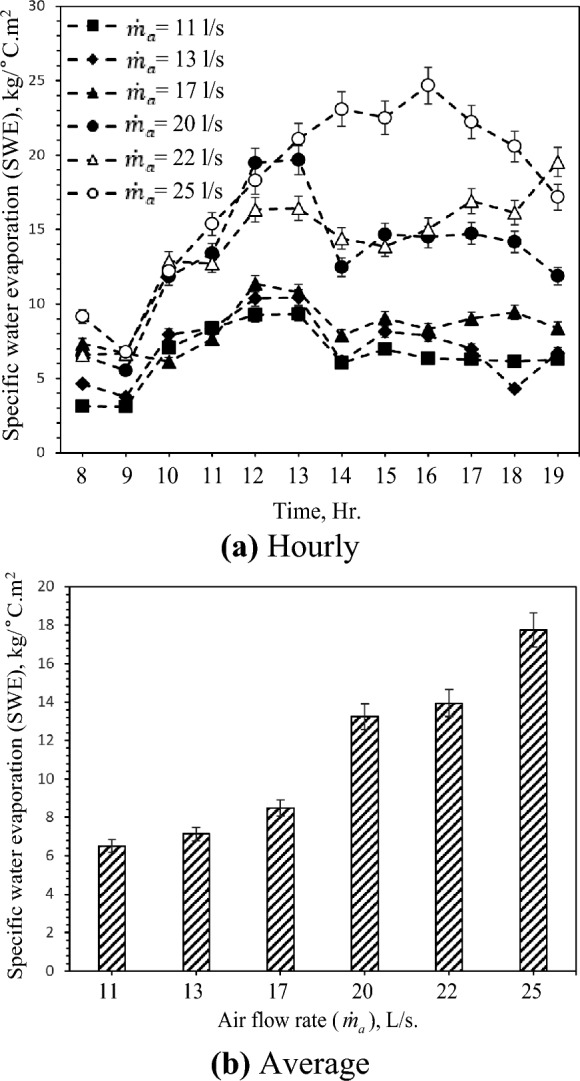
Hourly and average variation of specific water consumption with different air flow rate.

Specific water evaporation (SWE) is one of the evaporative coolers' performance measuring parameters. SWE is the porous clay vessel's evaporation rate per unit surface area divided by the mean temperature difference. Figure 9 shows the airflow rate, air temperature, and water temperature effects on SWE. As can be seen in Fig. 9a, SWE increases by increasing the inlet air temperature, and then SWE decreases with the increase of the inlet air temperature. This behavior is based on water evaporation rate and temperature difference with a constant surface area of the porous clay vessel. So, the two main factors that affect the SWE are air flow rate and solar radiation intensity due to their effects on water evaporation rate and temperature difference, respectively. Figure 9b shows that SWE increases with increasing the flow rate of air which is attributed to the relationship between water evaporation rate and cooling effect discussed in the previous sections. The highest and lowest SWE was found to be 17.8 kg/ °C.m^2^ and 6.5 kg/ °C.m^2^ for 25 L/s and 25 L/s air flow rate, respectively. SWE improves by 172.3% with rising 14 L/s in air flow rate and 1.7 L/hr water evaporation rate. Moreover, the maximum value of SWE (see Fig. 9a) is 24.7 kg/ °C.m^2^ at an air flow rate of 25 L/s.

### Specific cooling capacity (SCC)

**Figure 10:**
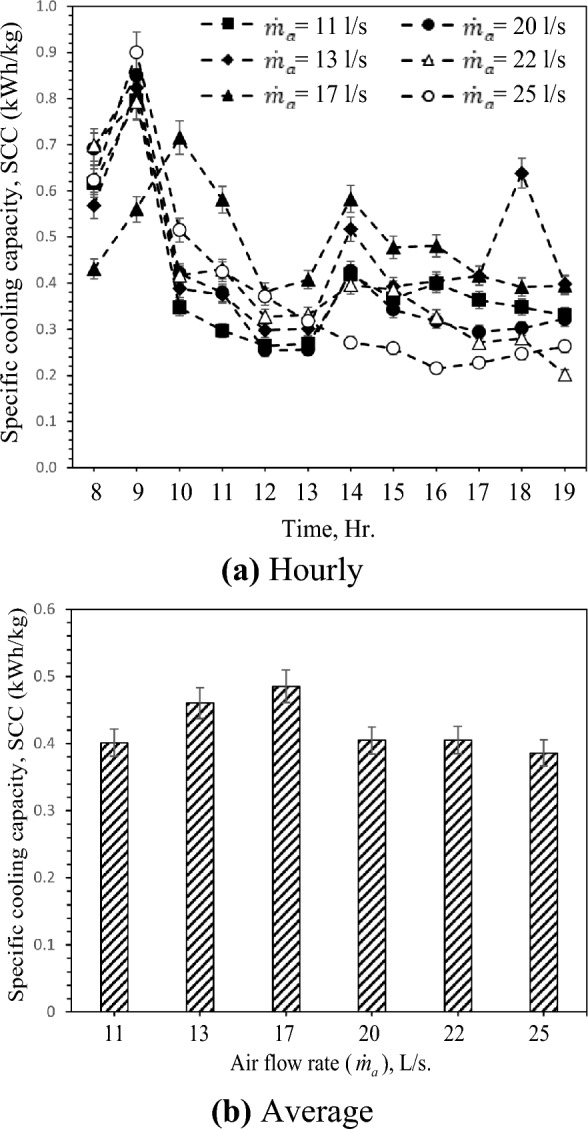
Hourly and average variation of specific cooling capacity with different air flow rate.

The variation of the specific cooling capacity (SCC) with various air flow rates is shown in Fig. 10. Figure 10a shows the decrease of SCC with the increase of inlet air temperature, respectively. As demonstrated in Fig. 10a, the highest and lowest values of SCC are 0.9 kWh/kg and 0. 2 kWh/kg at 25 L/s and 22 L/s, respectively. As shown in Fig. 10b, SCC increases with the increase of airflow rate until 17 L/s and then decreases to 25 L/s. This is attributed to the increase in air flow rate, which is more dominant than the decrease in air temperature difference and contact time between air and vessel surface due to the growth of air velocity. The reduction of SCC is due to the rise of the water evaporation rate, which has more effect on SCC than the effect of increasing cooling capacity. Furthermore, SCC increased by 20.9% by rising from 11 L/s to 17 L/s of air flow rate and then reduced by 20.4% by growing from 17 L/s to 25 L/s.

### Specific total cost (STC)

**Figure 11:**
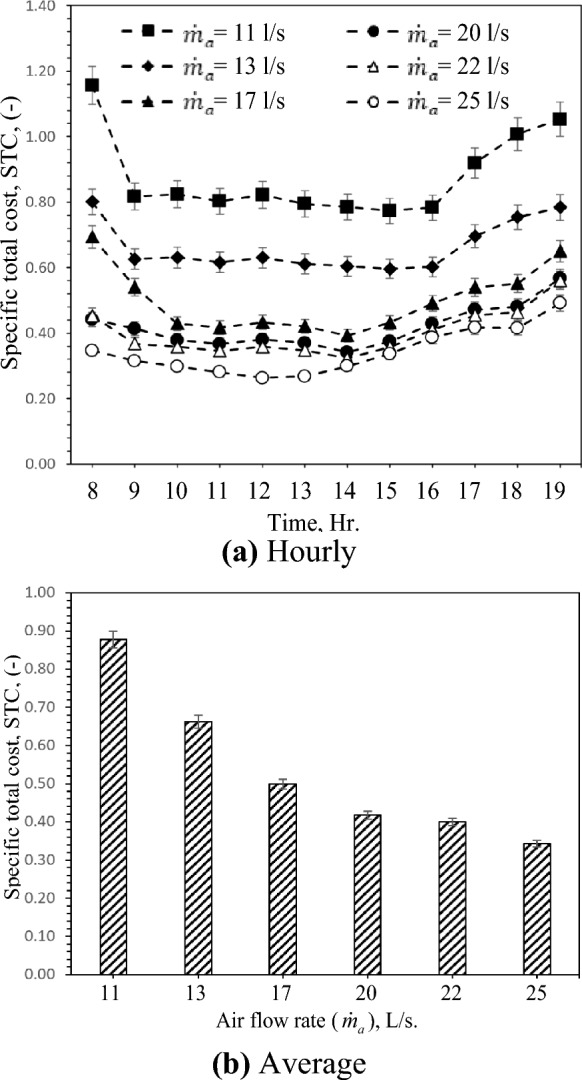
Hourly and average variation of specific total cost with different air flow rates.

The specific cost of the GAHE reduces with the increase of the inlet temperature conditions, as shown in Fig. 11. Figure 11a demonstrates that the airflow rate of 25 L/s at 12:00 has the cheapest specific cost, 0.26 USD/W. In this Figure, the parameter variation significantly affects the running and typical costs. The airflow rate increases the running cost (fan power cost) but decreases the regular cost. The cost of the GAHE with an airflow rate of 25 L/s is less than 11 L/s by about 61% due to the higher cooling capacity, as presented in Fig. 11b.

### Carbon dioxide emissions (CE)

**Figure 12:**
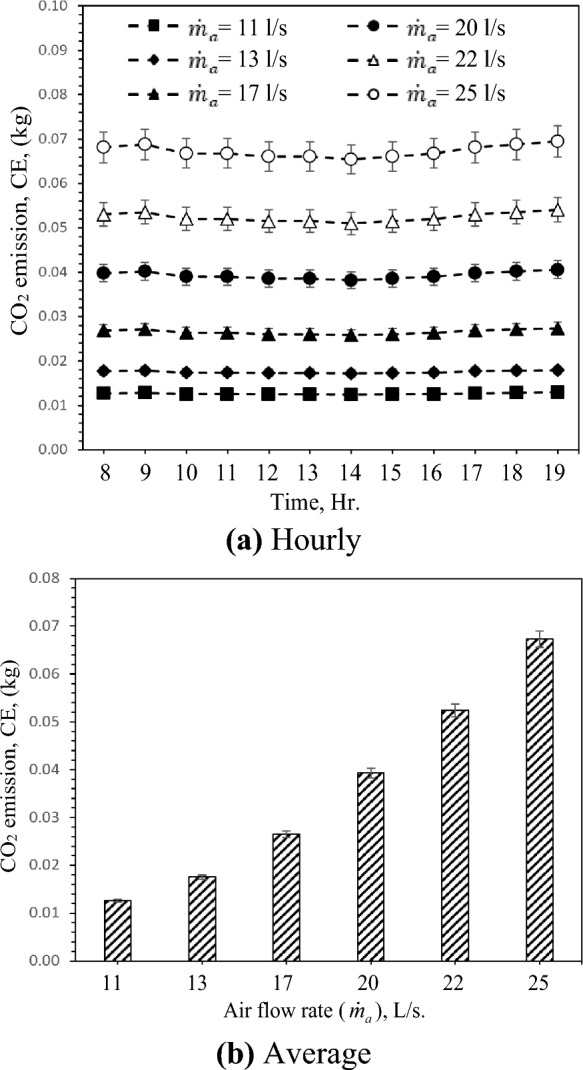
Hourly and average variation of CO_2_ emission (kg) with different air flow rate.

Emissions of carbon dioxide, or CO2 emissions, result from the combustion of fossil fuels to produce energy, such as electricity. The CO2 emission quantity is highly dependent on the operational and geometrical parameters of the GAHE. Figure 12 shows the hourly and average variation of CO_2_ emission (CE) with different air flow rates during the daytime. As presented in Fig. 12a, the CO_2_ emission does not fluctuate much with inlet temperature variation for different test cases. The GAHE will produce 0.07 kg CO_2_ if operated for one hour at 25 L/s air flow rate. As the air flow rate increases from 11 L/s to 25 L/s, the CO_2_ emission increases from 0.013 kg CO_2_ to 0.067 kg CO_2_ with a percentage of about 432.8%, as presented in Fig. 12b. Therefore, the increase in airflow dramatically raises CO2 emissions and negatively impacts the environment. The increased airflow rate improves cooling capacity but decreases EER, which has a negative impact on the environment.

In hot and arid climates, these results indicate that the GAHE is suitable for achieving an adequate supply temperature for space air conditioning. It is prevalent in the majority of Middle Eastern nations. Adopting porous clay vessels as moist media would provide additional reliability and integration-compatible advantages for building components.

### Exergy efficiency ratio

**Figure 13:**
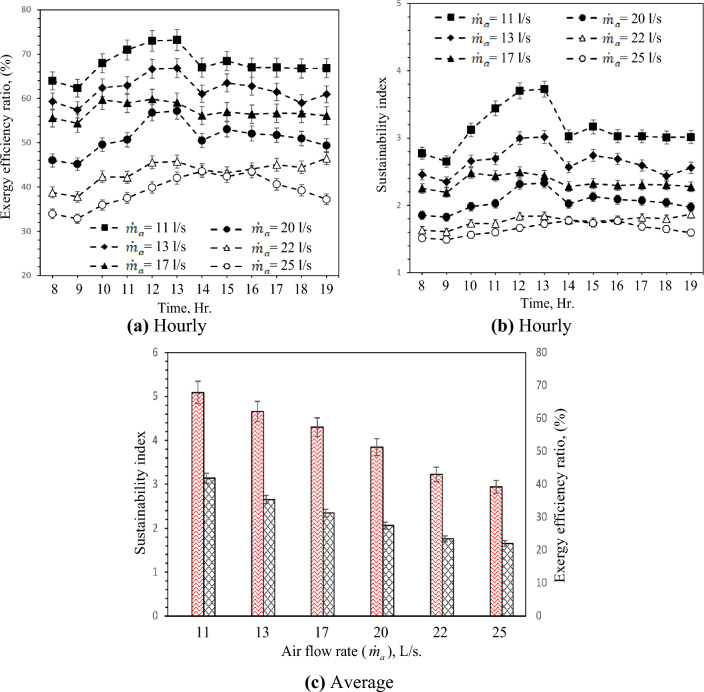
Hourly and average variation of exergy efficiency and Sustainability index with different air flow rates.

The hourly and average variation of exergy efficiency and sustainability index with different air flow rates are demonstrated in Fig. 13. Exergy efficiency is a viable approach to optimizing the utilization of energy resources, thereby enhancing efficiency and reducing parameters that adversely impact efficiency, such as irreversibility. Consequently, the system's efficiency and sustainability can be enhanced through the application of exergy efficiency^59^. As shown in Fig. 13a. The flow rate of 11 L/s achieved the highest exergy ratio of about 73% occurring at 13:00 and the lowest exergy ratio at a flow rate of 25 L/s. Also, the higher and lower sustainability index at flow rates 11 and 25 L/s, respectively, as indicated in Fig. 13b. This means the increment in airflow rate decreased the exergy ratio and sustainability index which has a negative impact on the environment. Figure 13c shows the average exergy ratio and sustainability index with different flow rates. The highest exergy ratio at flow rate of 11 L/s with increments of 11.29, 18.96, 32.2, 56.8, and 76.9% at flow rates of 13, 17, 20, 22, and 25 L/s, respectively. The highest sustainability index related to the highest exergy ratio at flow rate of 11 L/s with increments of 23.07, 33.30, 45.45, 77,77, and 88.23% at flow rates of 13, 17, 20, 22, and 25 L/s, respectively.

### Comparison of evaporative cooling materials and Earth-Air Heat Exchanger

The cooling capacity of the current proposed system was compared with similar systems, as indicated in Table 7. It is clear that the present system gives an acceptable outcome with a good performance.

**Table 7:** Comparison of evaporative cooling materials and Earth-Air Heat Exchanger.

Authors	Porous materials	Cooling effect, ΔT (°C)	Cooling capacity (kW)	Specific cooling capacity (kWh/kg)
Present work	Porous Clay Vessels	15.2	0.456	0.38
Laknizi^60^	Cellulosic pad	–	7-.4	–
Wijaksana et al.^61^	Gunny sack	4.6	4.6	–
Warke and Deshmukh^62^	Cellulose pad	2.7	1.6	–
Dogramacı et al.^63^	Eucalyptus fibers	11.3	0.6	–
Chen et al.^64^	Polymer hollow fiber	5.2	5	–

## Conclusion

The performance evaluation of the GAHE was evaluated experimentally under a wide range of ambient air temperature and RH based on the air-cooling effect, wet-bulb, and dew-point efficiencies, energy efficiency ratio, water evaporation rate, specific water evaporation, specific cooling capacity, specific total cost, and CO_2_ emission rate. The following is a summary of the current comprehensive parametric study's findings:

- The GAHE is an acceptable system that would achieve adequate supply temperature for space air conditioning in hot and dry climatic conditions.
- The increased airflow rate increases the cooling capacity but decreases EER, leading to a bad environmental effect.
- Increasing the air flow rate leads to an increase in the cooling capacity. Energy efficiency ratio (EER) reaches a maximum value of about 25.5 recorded at 15:00 PM with air flow rate = 11 L/s. The lowest value of EER is about 7.2, achieved when the inlet and outlet measured temperatures are the closest at 19:00 PM with air flow rate = 25 L/s.
- Increasing the air flow rate from 11 to 17 L/s increased the wet bulb efficiency, and then the airflow rate was inversely proportional to wet-bulb efficiency.
- The maximum and minimum average dew-point efficiency are 64% and 58% at 17 L/s and 22 L/s respectively.
- The water evaporation rate increases by 182.1%, increasing the air flow rate from 11 L/s to 25 L/s.
- The increase in the airflow rate increases the running cost (fan power cost) but decreases the total specific cost.
- The increment in airflow rate decreased the exergy ratio and sustainability index which has a negative impact on the environment

## Data Availability

The datasets used and/or analyzed during the current study available from the corresponding author on reasonable request.

## Appendix

All temperatures are directly measured, excluding the wet-bulb temperature, which can be obtained from Eq. (16)^65^;

15 $$\begin{array}{*{20}c} {{\text{T}}_{wb} = {\text{T}}^{*} {\text{tan}}^{ - 1} \left( {0.151977*(RH + 8.313659)^{0.5} } \right)} \\ { + {\text{tan}}^{ - 1} \left( {T + RH} \right) - {\text{tan}}^{ - 1} \left( {RH - 1.676331} \right)} \\ { + 0.00391838^{*} RH^{1.5} {\text{tan}}^{ - 1} \left( {0.023101^{*} RH} \right)} \\ { - 4.686035} \\ \end{array}$$

where T: Dry bulb temperature (C) and RH%: Relative humidity percentage (%).

The dew point temperature (Tdp) is when the air becomes saturated due to sensible cooling (i.e., without an increase in RH), and condensation occurs at constant pressure. It can be obtained by applying Eq. (17)^66^;

16 $$T_{dp} = 243.2\left[ {\frac{{\ln \left( {\frac{RH}{{100{\text{\% }}}}} \right) + \left( {\frac{17.62.T}{{243.12 + T}}} \right)}}{{17.62 - \ln \left( {\frac{RH}{{100{\text{\% }}}}} \right) - \left( {\frac{17.62.T}{{243.12 + T}}} \right)}}} \right]$$

Humidity ratio of humid air ($\omega )$ in g_w_/kg_da_^66^:

17 $$\omega = 216.7\left[ {\frac{{6.112 \times \frac{RH}{{100{\text{\% }}}} \times exp^{{\left( {\frac{17.62 \times T}{{243.12 + T}}} \right)}} }}{273.15 + T}} \right]$$

Dry air density ($\rho_{a}$) in kg/m^3^^67^:

18 $$\rho_{a} = 3.9147 - 0.016082T + 2.9013 \times 10^{ - 5} T^{2} - 1.9407 \times 10^{ - 8} T^{3}$$

Constant pressure specific heat for air ($C_{pa}$) in J/kg.K^68^:

19 $$C_{pa} = 1006\left( \frac{T}{293} \right)^{0.0155}$$

where (*T*) is the temperature in K.

## Latin symbols

$\dot{m}$: Mass flow rate (kg/s)
_*p*_: Pressure (N/m^2^)
T: Temperature (°C)
_W_: Power (W)
*E*: Energy (J)
*Cc*: Capital cost
$$f$$: Emission factor
*C*_*p*_: Specific heat (J/kg K)
*Q*: Heat (W)
*t*: Time (h)

## Greek symbols

*η*: Efficiency (dimensionless)
*ω*: Humidity ratio of humid air (g_w_/kg_da_)
*ρ*: Density (kg/m^3^)

## Subscripts

*a*: Air
$$dp$$: Dew point
*out*: Outlet
*in*: Inlet
*w*: Wall
*op*: Operation
*d*: Dray bulb
*wb*: Wet bulb
*c*: Sensible
*blower*: Blower

## Abbreviations

CE: Carbon dioxide emissions
EER: Energy efficiency ratio
GAHE: Ground air heat exchanger
PCV: Porous carbon vessele
RH: Relative humidity
*STC*: Specific total cost
SCC: Specific cooling capacity
SWE: Specific water evaporation
